# Role of Differential Signaling Pathways and Oxidative Stress in Diabetic Cardiomyopathy

**DOI:** 10.2174/157340310793566145

**Published:** 2010-11

**Authors:** Kenichi Watanabe, Rajarajan A Thandavarayan, Meilei Harima, Flori R Sari, Narasimman Gurusamy, Punniyakoti T Veeraveedu, Sayaka Mito, Wawaimuli Arozal, Vijayakumar Sukumaran, Arun Prasath Laksmanan, Vivian Soetikno, Makoto Kodama, Yoshifusa Aizawa

**Affiliations:** 1 Department of Clinical Pharmacology, Niigata University of Pharmacy and Applied Life Sciences, 265-1 Higashijima, Akiha-ku, Niigata City, Japan; 2 Cardiovascular Research Center, University of Connecticut School of Medicine, USA; 3 First Department of Medicine, Niigata University Graduate School of Medical and Dental Sciences, Niigata, Japan

**Keywords:** Diabetes mellitus, oxidative stress, cardiomyopathy, apoptosis, hypertrophy.

## Abstract

Diabetes mellitus increases the risk of heart failure independently of underlying coronary artery disease, and many believe that diabetes leads to cardiomyopathy. The underlying pathogenesis is partially understood. Several factors may contribute to the development of cardiac dysfunction in the absence of coronary artery disease in diabetes mellitus. There is growing evidence that excess generation of highly reactive free radicals, largely due to hyperglycemia, causes oxidative stress, which further exacerbates the development and progression of diabetes and its complications. Hyperglycemia-induced oxidative stress is a major risk factor for the development of micro-vascular pathogenesis in the diabetic myocardium, which results in myocardial cell death, hypertrophy, fibrosis, abnormalities of calcium homeostasis and endothelial dysfunction. Diabetes-mediated biochemical changes show cross-interaction and complex interplay culminating in the activation of several intracellular signaling molecules. Diabetic cardiomyopathy is characterized by morphologic and structural changes in the myocardium and coronary vasculature mediated by the activation of various signaling pathways. This review focuses on the oxidative stress and signaling pathways in the pathogenesis of the cardiovascular complications of diabetes, which underlie the development and progression of diabetic cardiomyopathy.

## INTRODUCTION

Cardiovascular disease represents the major cause of morbidity and mortality in diabetic patients [1]. Accumulating data from experimental, pathological, epidemiological, and clinical studies have shown that diabetes mellitus results in cardiac functional and structural changes, independent of hypertension, coronary artery disease, or any other known cardiac diseases, which support the existence of diabetic cardiomyopathy [2-4]. In 1972, Rubler *et al*. [5] first described a specific type of cardiomyopathy related to diabetes, suggesting that this myocardial disease exists as an independent clinical entity. The term "diabetic cardiomyopathy" was therefore proposed. Diabetic cardiomyopathy, as an early complication of diabetes, is manifested by diastolic dysfunction followed by abnormalities in systolic function [6]. When presenting with other cardiovascular complications (i.e., ischemic heart disease or hypertension), diabetic patients have a much worse prognosis than non-diabetic patients and are more prone to progress to congestive heart failure [7]. The underlying diabetic cardiomyopathy appears to contribute to accelerated heart failure [1].

Under physiological conditions, reactive oxygen species (ROS), such as superoxide radical, hydroxyl radical, and hydrogen peroxide (H_2_O_2_) are continuously produced in many cells, but ROS levels are regulated by a number of enzymes and physiological antioxidants, such as superoxide dismutase, glutathione peroxidase, catalase, and thioredoxin. However, when the production of ROS becomes excessive, oxidative stress will develop and impose a harmful effect on the functional integrity of biological tissue. Large experimental and clinical studies have shown that the generation of ROS is increased in both types of diabetes and that the onset of diabetes and its complications, including diabetic cardiomyopathy, are closely associated with oxidative stress [8, 9]. The production of ROS has been shown to be increased in patients with diabetes [10-14], and increased ROS production may be involved in the onset or development of diabetic vascular complications. It has been postulated that hyperglycemia, a key clinical manifestation of diabetes, may produce ROS through the formation of advanced glycation end products (AGEs) [11, 12] and altered polyol pathway activity [13], and through the activation of NADPH oxidase *via* protein kinase C (PKC) [15]. 

Hyperglycemia-induced oxidative stress is a major risk factor for the development of micro-vascular pathogenesis in the diabetic myocardium, which results in myocardial cell death, hypertrophy, fibrosis, abnormalities of calcium homeostasis, and endothelial dysfunction [16-18]. Although these pathogenic factors cause diabetic cardiomyopathy, probably *via* a different mechanism [16, 19-23], their major contribution to diabetic cardiomyopathy is oxidative stress [24], which is derived directly from these pathogenic factors or indirectly from metabolic intermediates caused by these factors, such as the formation of AGEs and production of cytokines or peptides, such as angiotensin II (AT-II). 

Myocardial cell death, hypertrophy and fibrosis are the most frequently proposed mechanisms to explain cardiac changes in diabetic cardiomyopathy. Nevertheless, the signaling pathways that regulate diabetic cardiomyopathy have not been fully elucidated. This review focuses on emerging evidence on oxidative stress and signaling pathways in the pathogenesis of the cardiovascular complications of diabetes, which underlie the development and progression of diabetic cardiomyopathy. 

## DIABETES INDUCTION BY STREPTOZOTOCIN

Streptozotocin (STZ) is a glucosamine-nitrosourea compound that shows selective cytotoxicity to pancreatic β cells and is used as an agent to induce experimental animal diabetes. STZ is injected at a dose from 150 to 200 mg/kg body weight (BW) in mice. In our study, type-I diabetes was induced in mice by a single i.p injection of STZ at a dose of 150 mg/ kg BW [25, 26]. STZ action on beta cells is accompanied by characteristic alterations in blood insulin and glucose concentrations. Two hours after injection, hyperglycemia is observed with a concomitant drop in blood insulin. About six hours later, hypoglycemia occurs with high levels of blood insulin. Finally, blood insulin levels decrease and hyperglycemia develops. STZ is taken up by pancreatic β cells *via *glucose transporter (GLUT)2. Intracellular action of STZ results in changes in DNA in pancreatic β cells causing its fragmentation. Recent experiments have proved that the main reason for STZ-induced β cell death is alkylation of DNA. The alkylating activity of STZ is related to its nitrosourea moiety, especially at the O6 position of guanine. Since STZ liberates nitric oxide (NO) when STZ is metabolized inside cells, NO causes DNA damage in pancreatic islet cells; however, the results of several experiments provide evidence that NO is not the only molecule responsible for the cytotoxic effect of STZ. STZ was found to generate ROS, which also contribute to DNA fragmentation and evoke other deleterious changes in cells. Augmented ATP dephosphorylation increases the supply of substrate for xanthine oxidase (β cells possess high activity of this enzyme) and enhances the production of uric acid – the final product of ATP degradation. Then, xanthine oxidase catalyses the reaction in which the superoxide anion is formed. As a result of super-oxide anion generation, hydrogen peroxide and hydroxyl radicals are formed [27]. 

It can be stated that potent alkylating properties of STZ are the main reason for its toxicity; however, the synergistic action of both NO and ROS may also contribute to DNA fragmentation and other deleterious changes caused by STZ. NO and ROS can act separately or form highly toxic peroxynitrate (ONOO); therefore, intracellular antioxidants or NO scavengers substantially attenuate STZ toxicity. STZ-induced DNA damage activates poly ADP-ribosylation. This process leads to the depletion of cellular NAD+, further reduction of ATP content, and subsequent inhibition of insulin synthesis and secretion [27].

## DIASTOLIC DYSFUNCTION

Changes in diastolic dysfunction are a widely reported finding in diabetic animals [28, 29], and patients without evidence of heart disease caused by other factors [30, 31]. The presence of diastolic dysfunction in diabetic hearts may relate to uncoupling of the contractile apparatus (which drives early relaxation), without concomitant increases in chamber stiffness (which produces more late diastolic changes) [32]. Moreover, isoproterenol administration to hearts from 4-week-old diabetic rats reduced the peak rate of relaxation, although the rate of contraction increased normally [33]. Diastolic functional parameters in diabetic patients are analogues to those in animal studies, where the left ventricular (LV) diastolic dysfunction appears to be quite common in well-controlled type-II diabetic patients without clinically detectable heart disease [34]. 

## SYSTOLIC DYSFUNCTION

Animal studies have shown that diabetes is also associated with systolic dysfunction [35-37]. Similar findings were reported in intact animals; heart rate, systolic blood pressure, and fractional shortening were significantly reduced in diabetic animals compared with control animals [38]. Although a number of studies have confirmed the association of LV systolic dysfunction with diabetes mellitus, this finding has not been uniformly reported [39-42]; however, many patients who have normal LV systolic function at rest may show abnormalities during exercise or dobutamine stress [39, 43] indicating that LV systolic reserve is reduced in those patients.

## MYOCARDIAL STRUCTURAL CHANGES

A number of studies in both animals and humans have shown structural changes in parallel with functional changes in heart of diabetic disease, in the absence of hypertension, coronary artery disease, or intraventricular conduction defects [44-48]. The most prominent histopathological finding in diabetic patients and animals is fibrosis, which may be perivascular, interstitial or both [40, 49, 50]. 

## METABOLIC DISTURBANCES

A significant reduction in myocardial glucose supply and utilization has been observed in isolated diabetic cardiomyocytes [51] and diabetic patients [52]. A slow rate of glucose transport across the sarcolemmal membrane into the myocardium, probably due to the cellular depletion of GLUTs 1 and 4, mainly restricts glucose utilization in the diabetic heart [53, 54]; however, this can be corrected by insulin therapy [54, 55] and GLUT-4 overexpression [56]. A second mechanism of reduced glucose oxidation is *via* the inhibitory effect of fatty acid oxidation on pyruvate dehydrogenase complex due to high circulating free fatty acid (FFA) [57].

Elevated FFA levels are believed to be one of the major contributing factors in the pathogenesis of diabetes [58, 59]. Elevation of circulating FFAs is caused by enhanced adipose tissue lipolysis, and high tissue FFAs are caused by the hydrolysis of augmented myocardial triglyceride stores. Moreover, high circulating and cellular levels of FFAs may result in abnormally high oxygen requirements during FFA metabolism and the intracellular accumulation of potentially toxic intermediates of FFA, all of which lead to impaired myocardial performance and severe myocardial changes [58, 59]. Furthermore, the availability of carnitine, an essential substrate for myocardial FFA metabolism, is usually reduced in diabetes [60]. 

Several sarcolemmal changes have been identified during diabetes which includes alterations in sarcolemmal calcium binding [61], Na^+^-K^+^ ATPase [62], and calcium pump activity [63]. Calcium transport by the sarcoplasmic reticulum (SR) is another major mechanism by which myocardial levels of calcium, and thereby tension development, are modulated. In diabetic hearts, SR Ca^2+^ binding, Ca^2+^-Mg^2+^ ATPase activity are decreased, leading to a defect in SR Ca2^+^ transport [64], which then correlates with slower relaxation [65]. SR Ca^2+^ ATPase activity and calcium pump protein (SERCA2a) are reduced in diabetic hearts [66]. Abnormal systolic and diastolic functions were normalized after overexpression of SERCA2a in STZ-induced diabetic rat hearts [67]. A number of studies have shown that Ca^2+^ ATPase activities of myosin and actinomyosin are depressed, thus accounting for decreased shortening velocity of the cardiac muscle, which is associated with a myosin isoenzyme shift from the more active V1 isoform to the less active V3 isoform [68]. In addition, mitochondrial oxidative capacity, Mg^2+^ ATPase activity, and Ca^2+^ uptake activity are all depressed in the diabetic myocardium [69]. The above changes likely result from the accumulation of toxic molecules, such as long-chain acyl carnitines, free radicals, and abnormal membrane lipid content. Importantly, in several studies, abnormalities in metabolism and hemodynamics in diabetic animals were reversed by both islet transplantation and insulin therapy [70]. 

## MYOCARDIAL APOPTOSIS IN DIABETES

Apoptosis is a tightly regulated mechanism for eliminating damaged or superfluous cells without harming their healthy neighbors. Controlled deletion of cells serves many useful functions in development and during stress to ensure the survival and integrity of the organism; however, when apoptosis is not balanced by cell replacement in the adult myocardium, functional impairment can occur. Cardiomyocytes possess the necessary apparatus for cellular suicide and activate the process in response to a range of stresses, including hypoxia, free radical stress, viral infection, adrenergic overstimulation, and work overload. Apoptosis occurs concomitantly with necrosis in the infarcted and reperfused myocardium [71], end stage heart failure [72], postinfarction LV remodeling [73], diabetes [74], and during the regression of hypertrophy [75].

Several studies have indicated that diabetes induces myocardial apoptosis in human patients [76] as well as in diabetic animal models [77-79]. STZ-induced diabetic rats and mice showed increased myocardial apoptosis on day 3-14 and decreased on day 28 after STZ treatment [77-79]. It will be interesting to know whether myocardial apoptosis is specifically diabetes-dependent. Cardiac specimens from diabetic patients without hypertension showed an increase in myocardial apoptosis relative to non-diabetic patients [76]. However, no difference in myocardial cell death was observed between diabetic patients with hypertension and diabetic patients without hypertension [76]. These results indicate that myocardial apoptosis in diabetic patients is directly related to diabetes, but not to coexisting hypertension. The possibility that myocardial apoptosis secondary to the application of STZ can be ruled out from the study of Cai *et al*. [79], who found no significant increase in myocardial apoptosis in the STZ-treated mice without hyperglycemia. Moreover, diabetic mice treated with insulin showed reduced elevation of blood glucose levels and inhibition of myocardial apoptosis on day-3 after STZ treatment [79]. Since dehydration is a common phenomenon in diabetic subjects, dehydration-induced myocardial apoptosis can be excluded from the study [77], where both diabetes and restriction in food intake decreased BW and heart weight, leading to a modest depression of cardiac function, but only diabetic hearts showed an increase in myocardial apoptosis. These results indicated that myocardial apoptotic cell death was directly related to diabetic pathogenesis, not secondary to the application of STZ, and hypertension and dehydration coexisted with diabetes. In addition, several *in vitro* studies have indicated that a high level of glucose induces apoptosis in cultured adult cardiomyocytes, smooth muscle, and endothelial cells [79].

## MYOCARDIAL HYPERTROPHY AND FIBROSIS 

The mammalian myocardium undergoes a period of hypertrophic growth during postnatal maturation, which is characterized by an increase in the size of individual cardiac myocytes without cell division [80]. The pattern of developmental hypertrophy is reinitiated in the adult heart in response to diverse mechanical, hemodynamic, hormonal, and pathologic stimuli [81]. Increased mechanical and neurohumoral load, such as hypertension, ischemic heart disease, valvular insufficiency, and cardiomyopathy, increases wall thickness and results in concentric hypertrophy [80]. At the cellular level, cardiac myocytes respond to diverse types of biomechanical stress by initiating several different processes that *via* the activation of transcription factors lead to hypertrophic gene expression and growth of individual myocytes. Initially, the response is beneficial, but when prolonged, it leads to pathological myocyte hypertrophy. The first genetic response to increased load is activation of a pattern of early response, or immediate early genes: *c-fos*, *c-myc* and *c-jun* [82]. This is followed by the induction of certain genes, such as atrial natriuretic peptide (ANP), brain natriuretic peptide (BNP), β –myosin heavy chain (MHC) and α-skeletal actin (SkA), accompanied by the development of a hypertrophic phenotype characterized by an increased cell-surface area, protein concentration and protein to DNA ratio [83]. 

Myocardial fibrosis can occur in patients who have hypertrophic cardiomyopathy in the absence of epicardial coronary disease [84]. Fibrosis is attributed to replacement fibrosis caused by focal myocyte necrosis [85, 86] and increased interstitial fibrosis, in part due to the reaction of connective tissue cells to pathological loads [86]. Myocardial fibrosis and cardiac hypertrophy are the most frequently proposed mechanisms to explain cardiac changes in diabetic cardiomyopathy [50]. Indeed, diabetic heart disease may simply reflect increased interstitial fibrosis in the heart, because collagen accumulation occurs mainly as a result of an increase in type III collagen in the diabetic heart [87]. Transforming growth factor (TGF)β1 is present in both cardiomyocytes and myocardial fibroblasts [88]. In the heart, TGFβ1 has been shown to be expressed at high levels during cardiac development [89] and pathology [90].

## SIGNALING PATHWAYS IN CARDIAC APOPTOSIS, HYPERTROPHY AND FIBROSIS

Hyperglycemia/diabetes-induced changes in myocardial structural and functional properties are mediated through the activation of various signaling pathways, e.g. *via* diacyl glycerol (DAG)-induced activation of PKC [91], ROS [92], AT-II [93], TGFβ1 [94] etc. Recently, the involvement of various mitogen activated protein kinase (MAPK) activities has been characterized in different diabetic tissues, such as glomerular mesangial cells [95], human umbilical vein endothelial cells (HUVEC) [96], bovine pulmonary artery endothelial cells (PAEC) [97], and dorsal root ganglion [98]; therefore, the specific role of MAPKs in the diabetic myocardium cannot be excluded.

## MAPK SIGNALING

There are multiple MAPK pathways in all eukaryotic cells, which allow the cells to respond differently to divergent inputs. MAPK signaling cascades are usually divided into three parallel pathways: extra cellular signal regulated kinase (ERK), c-jun NH_2_ kinase (JNK) and p38 MAPK pathways. All MAPK pathways include three signaling levels, i.e. MAPK kinase kinase kinase (MAPKKK) activating, MAPK kinase kinsae (MAPKK), which in turn activates MAPK. Activation of MAPKKKs results from translocation, oligomerization, and phosphorylation by upstream kinases [99, 100]. Active MAPKKKs phosphorylate serine and threonine residues in MAPKKs, which in turn activate tyrosine and threonine residues in the activation loop of MAPKs. Most physiological substrates of MAPKs possess specific binding sites for MAPKs that allow strong interactions with selectivity for MAPK subfamilies [101]. MAPKs also possess complementary docking sites, which allow them to interact with MAPK binding domains on substrate proteins [102]. The signaling mechanism is coordinated by the interaction of components of the protein kinase cascade with scaffold proteins, such as JNK interacting protein-1 and MEK1 partner [103]. 

## JNK SIGNALING

Cardiomyocyte apoptosis can be stimulated by various factors, such as oxygen radicals, cytokines, autocoids, and sphingolipid metabolites, various physical and chemical stresses [76-79]. Diabetes-induced cardiomyocyte apoptosis was found in human patients [76] and various animal models [77-79]. MAPK family members play an important role in cardiac survival signaling [104], and modulation of MAPK activities has been characterized in various diabetic tissues [95-98]. We found enhanced activation of JNK and a higher percentage of apoptosis 3 and 7 days after diabetes induction in mice [25]. Also, activation of JNK and apoptosis in the diabetic myocardium were significantly correlated (*P *< 0.0001, r = 0.9472). This study indicates the possible involvement of the JNK pathway in mediating early stage diabetes-induced cardiomyocyte apoptosis in mice. Several other studies have reported that JNK activity leads to cardiomyocyte apoptosis. For example, oxidative stress-induced cardiomyocyte apoptosis is mediated by the activation of JNK, which directly activates the mitochondrial death mechanism, and β-adrenergic receptor-stimulated apoptosis in cardiac myocytes is mediated by JNK-dependent activation of mitochondrial pathway [105]. High glucose-induced apoptosis in HUVEC is mediated by the activation of JNK and caspase-3 [96]. Some studies have demonstrated that JNK induction appears to be upstream of interleukin-1β converting enzyme (ICE/CED-3) proteases in apoptosis induced by ultraviolet C and α-radiation [106] and anticancer drugs [107], whereas others documented the activation of JNK downstream of ICE/CED-3 proteases in the CD95 pathway [108]. 

## p38 MAPK SIGNALING

High glucose or diabetes induced the activation of p38 MAPK in 1- and 2-month diabetic glomeruli [109], Recently, in STZ-induced diabetic myocardium, p38 MAPK activation was found to be decreased 7 days after STZ injection [110]. Very recently, it has been reported that 7 days after STZ injection phosphorylated p38 MAPK was decreased in the diabetic rat myocardium when compared to non-diabetic rats [110]. In our study, we found enhanced activation of p38 MAPK 1, 28 and 56 days after STZ injection in mice [25, 26]. These results support earlier studies, in which p38 MAPK activity was significantly increased in one and two month diabetic glomeruli [109], and STZ-induced diabetes in rats activated p38 MAPK, resulting in the phosphorylation of heat shock protein 25 [111]. In contrast, high glucose did not activate p38 MAPK in HUVEC [96] and in bovine PAEC [97]. These discrepancies in the role of p38 MAPK in high glucose diabetes may be partly explained by the difference in experimental models, presence of multiple p38 MAPK isoforms (p38α, p38β, p38γ and p38δ, and the existence of several MAPKK, which activate p38 MAPK; however, the role p38 MAPK in diabetic myocardium is not clear. Moreover, different studies indicate that the activation of p38 MAPK can be ascribed either a pro-apoptotic [112] or anti-apoptotic role [113], depending on the type of stimulus in cardiac myocytes. In our study [25], no significant correlation between p38 MAPK activation and apoptosis was found, but, the percentage of cardiomyocyte apoptosis was decreased when p38 MAPK activation was increased 1, 28 and 56 days after diabetes induction, and an increase in apoptosis was found when p38 MAPK activation was minimal 3 and 7 days after diabetes induction. Therefore, from our results, it can be speculated that p38 MAPK may play an anti-apoptotic and/or apoptotic role in diabetes-induced cardiomyocyte apoptosis. 

## ERK1/2 SIGNALING

Enhanced phosphorylation of ERK was found in the lumbar dorsal ganglia of diabetic rats [98] and markedly increased phosphorylation of ERK1/2 was found in the myocardium of diabetic rats [110]. As demonstrated in other studies [96, 97], we found that ERK1/2 were not activated during the course of diabetes in mice [25]. Recently, Zhang *et al*. reported that activated p38 MAPK directly interacts with ERK1/2 and blocks their phosphorylation by MAPK/ERK kinase1 (MEK1) [114] .

## APOPTOSIS SIGNAL REGULATING KINASE (ASK) 1 SIGNALING

ASK1, a MAPKKK, is involved in biological responses such as apoptosis, inflammation, differentiation and survival in different cell types. Activated ASK1 relays signal to JNK and MAPK [115, 116]. We have recently reported that ASK1 activity was associated with de-phosphorylation at phospho-ASK1 (Ser-967), which has been identified as the 14-3-3 protein binding site for ASK1 in the diabetic myocardium [117]. High glucose-induced apoptosis in HUVEC is mediated by the activation of JNK and caspase-3 [96]. Moreover, we previously showed enhanced activity of the downstream effector of ASK1, JNK, and a higher percentage of apoptosis in mice 3 days after diabetes induction relative to normal mice [25, 117]. Hence we proposed that diabetes-induced ASK1 activity mediates myocardial apoptosis by downstream activation of JNK.

## PKC SIGNALING

Cardiovascular tissues showed the up regulation of PKC activities in diabetic state. PKC activity was significantly increased in the membrane fraction of diabetic hearts compared with controls, and the increased activity was accompanied by a decrease in cytosolic PKC activity in these diabetic hearts [118]. The increase in membrane-bound PKC activity thus appears to be due to translocation of the enzyme from the cytosolic to membrane fraction. These results indicate that the development of diabetic cardiomyopathy is accompanied with a high membrane-bound PKC level [118]. This is probably caused by the de novo synthesis of DAG in response to overflow of the glycolysis pathway in hyperglycemic conditions [119]. Accumulation of metabolites in the glycolysis pathway, such as glyceraldehyde 3-phosphate, will drive the synthesis of DAG, which in turn recruits primed PKC into the plasma membrane to render a competent kinase, a key event in its activation [119]. The level of DAG content in the diabetic heart is positively correlated with the blood glucose levels [120]. In the diabetic heart, PKCβ2 has been preferentially found to be activated in the heart and aorta of diabetic animals [91, 120, 121]. Guo *et al*. [121] found that PKCβ2 is significantly upregulated in the diabetic heart at both the transcriptional and translational levels. Moreover, targeted overexpression of PKCβ2 in the mouse myocardium resulted in LV hypertrophy, fibrosis, and decreased LV performance, similar to diabetic cardiomyopathy [122, 123]. Ventricles from patients with end-stage heart failure show increased expression of PKCβ2 and increased membranous PKC activity within cardiac myocytes [124]. Oral administration of a PKCβ-specific inhibitor normalizes blood flow and vascular barrier function in several organs of diabetic animals [125-127]. Although it is generally agreed that PKC activation contributes to cardiovascular dysfunction [128, 129], no consensus has been reached regarding the molecular basis of PKC upregulation in hyperglycemia or diabetes. We [26] examined the role of PKCβ2 in the diabetic myocardium of mice, and found that PKCβ2 was significantly increased 28 and 56 days after STZ injection in the LV cytosolic lysate of mice compared to control mice. The correlation coefficient (r) values obtained between the level of expression of PKCβ2 and cardiac cell size, and between the level of expression of PKCβ2 and fibrosis were higher in diabetic mice. 

## GLYCOGEN SYNTHASE KINASE (GSK) 3β SIGNALING

Recently, we have reported [130] that GSK3β is up-regulated in the early stage of STZ-induced diabetic myocardium relative to normal. Activation of caspase 3 is reported to be a downstream event in GSK3β signaling in mediating myocardial apoptosis [131]. The expressions of activated caspase 3 and myocardial apoptosis were significantly increased 3 days after STZ injection, and correlated with the enhanced activation of GSK3β and it was suggested for the first time that GSK3β plays an important role in mediating myocardial apoptosis in the diabetic myocardium. Previously, we have shown that the activation of JNK was correlated with myocardial apoptosis [25]. Very recently, Enguita *et al*. [132, 133] reported that JNK as well as GSK3β signaling pathways may mediate cell death pathways; therefore, it was worth examining the roles of GSK3β and JNK in mediating myocardial apoptosis in the diabetic myocardium. GSK3β is shown to inhibit cardiac hypertrophy by preventing the nuclear translocation of nuclear factor of activated T-cells (NFATc3) *via* the phosphorylation of NFATc3 [134]. NFATc3 is a transcription factor which activates several genes related to cardiac hypertrophy [135]. We found that the phosphorylation of GSK3β and the nuclear translocation of NFATc3 were elevated in mice 28 days after STZ injection, without a significant alteration in AKT activity, suggesting that there are other possible upstream activators of GSK3β that control its signaling in the diabetic myocardium, converting the pro-apoptotic stimuli of GSK3β observed in early stages of the disease [130]. Recently, GSK3β was also shown to be inactivated by PKC [136], and several studies and our study also indicated that PKCβ2 isoform is elevated in the diabetic myocardium [26, 130]. It is intriguing to note a parallel relationship between PKCβ2 and the enhanced phosphorylation of GSK3β ser 9 (indicator of inactive GSK3β) activity, which speculate that PKCβ2 may negatively regulate GSK3β, which may further activate the nuclear translocation of NFATc3 and the transcription of cardiac hypertrophic genes in the diabetic myocardium. Future studies addressing the relationship among PKCβ2, GSK3β and p38 MAPK may reveal interesting findings in the diabetic myocardium.

## ROLE OF OXIDATIVE STRESS AND AT-II IN THE PATHOGENESIS OF DIABETIC CARDIOMYOPATHY

Mitochondrial damage is related to ROS formation and plays an important role in the development of diabetic cardiomyopathy [137]. The increase in ROS serves to decrease the antioxidant capacity of the diabetic myocardium, contributing significantly to oxidative stress and the resultant myocardial damage. This damage causes cardiac morphological and functional abnormalities. Oxidative stress has recently been proposed as the unifying factor for the damaging effect of hyperglycemia [138, 139]. Cell death is an important determinant of cardiac remodeling because it causes a loss of contractile units, compensatory hypertrophy of myocardial cells and reparative fibrosis [140]. Apoptotic cell death associated with increased oxidative stress in multiple organ systems of diabetes mellitus has been well documented [141, 142]; however, the precise mechanism(s) by which ROS accumulation leads to compromised heart function and the effect of antioxidant therapy in diabetic subjects is largely unknown. Therefore, it is important to study the signaling pathways and molecular mechanisms by which hyperglycemia-induced (or, presumably, STZ-induced) oxidative stress leads to cell death and myocardial pathogenesis.

Several sources have been proposed for enhanced ROS formation in hyperglycemia. The renin-angiotensin system (RAS) is known to play a major role in the regulation of blood pressure and other functions of the cardiovascular system [143]. Hyperglycemia activates the local RAS, resulting in the formation of AT-II, and it has been shown clinically and experimentally that AT-II induces oxidative damage by producing ROS through the NADH/NADPH oxidase system [76-78, 134]. Such effects of AT-II are mediated through AT1 receptor in the heart [76]. The contribution of RAS has been implicated in the pathogenesis of diabetic cardiomyopathy [144, 145]. Up-regulation of the local RAS in diabetes may enhance oxidative damage, activating cardiac cell apoptosis, and necrosis [76].

AT-II is a multifunctional hormone that regulates many important cellular processes, including vascular function, cell growth, apoptosis, migration, fibrosis and inflammation, [146]. AT-II elicits its actions *via* two distinct receptors; AT1 and AT-II type 2 (AT2) receptors [146]. Most known physiological and pathophysiological effects of AT-II are mediated *via* AT1 receptors. Current research in the field of vascular biology demonstrates that AT1 receptor activation stimulates non-phagocytic NADPH oxidase and the generation of •O_2_ **– **in various cell types [147, 148]. AT-II-mediated superoxide generation is upregulated in diabetes, hypertension and atherosclerosis, and is involved in redox-dependent signaling cascades [147]. The increased AT-II receptor levels in the STZ-induced diabetic heart, and the number of AT-II receptor sites per myocyte paralleled the change in myocyte apoptosis [77]. 

The increase in interstitial fibrosis in the diabetic heart is mainly due to the accumulation of type III collagen [87]. The effects of AT-II may also be promoted by the production and release of TGFβ1 by cardiac fibroblasts [149]. Treatment with an AT1 receptor antagonist has been demonstrated to prevent interstitial fibrosis in the LV [150]. Myocardial remodeling such as decreased myofibrillar Ca^2+^-ATPase, Mg^2+^-ATPase and myosin ATPase activities were seen in diabetic animals, and were reversed by treatment with AT1 receptor blocker [151]. Also, it has been shown that in STZ-induced diabetic rats, the loss of β-adrenergic-mediated enhancement of glucose uptake and abnormal electrophysiological properties, such as the prolongation of action potential duration and decrease in transient outward current (Ito), were restored after treating animals with AT1 receptor blocker [93]. In our study, treating STZ-induced diabetic mice with an AT1 receptor blocker attenuated diabetes-induced cardiac hypertrophy and fibrosis, indicating that diabetes-induced cardiac hypertrophy and fibrosis signaling is mediated through the AT1 receptor. Also, we found that treatment with AT1 receptor blocker reduced diabetes-indu-ced cardiac expressions of ANP, TGFβ1 and collagen III [130]. 

Blockade of AT1 receptor decreased perivascular fibrosis in obese mice [152] and OLETF rats [153]. The beneficial effects of AT1 receptor blockade in diabetic cardiomyopathy were explained through the activation of GLUT 4 [154], and peroxisome proliferator activated receptor (PPAR) γ [155]. Previously, we have shown that diabetes-induced cardiac hypertrophy and fibrosis are exacerbated in mice 28 days after STZ injection [26], where PKCβ2 increased in the LV tissue; however, the upstream signaling during diabetes-induced cardiac hypertrophy and fibrosis is not known.

Nishikawa *et al*. have shown that hyperglycemia increases ROS production, inducing oxidative damage, which in turn activates the death pathways leading to complications of diabetes [139]. Glucose autoxidation and protein glycation are both processes that are accelerated by high glucose and produce superoxide radical or other ROS [156]. NADPH oxidase has been shown to be stimulated by hyperglycemia in several cell types [157], and its mRNA in endothelial cells is induced 7-fold by high glucose [158]. High glucose can turn on NADPH oxidase by de novo synthesis of DAG [159], which activates PKC, which in turn can activate NADPH oxidase by phosphorylation of p22 phox and p47 phox [160]. In addition to mitochondrial sources of ROS, superoxide anion can be derived from NO synthase [161] and NADPH oxidases [134, 148]. Recently, it has been shown that membrane-associated NADPH oxidases are the primary physiological producers of superoxide in several animal models including diabetes [162]. Also, a high glucose level stimulates ROS production through PKC-dependent activation of NADPH oxidases in smooth muscle cells and endothelial cells [159]. 

On the other hand, high glucose can turn on the activation of p22 phox, a component of NADPH oxidase by de novo synthesis of DAG [159], which can further activate PKC [163]. Moreover, it has been shown that AT-II-induced NADPH oxidase is partly mediated through PKC [163]; therefore, the possibility of indirect control of oxidative stress in GSK3β through PKC also exists in the diabetic myocardium. In our study, myocardial AT-II and oxidative stress are elevated in the diabetic myocardium [130]. 

Recently, we have reported treatment with an AT1 receptor blocker as well as the antioxidant reduced the oxidative stress, suggesting that these events are essential for the development of diabetic cardiomyopathy, and their inhibition improves molecular and pathological events in the diabetic myocardium [130]. Tempol, a super oxide dismutase mimetic, by blocking AT1 receptor and reducing AT-II levels, resulted in almost identical reduction of active GSK3β/JNK-induced caspase-3 signaling 3 days after STZ injection. Also, treatment with either AT1 receptor blocker or tempol attenated STZ-induced cardiac hypertrophy and fibrosis by attenuating the inactivation of GSK3β and thus preventing the nuclear translocation of NFATc3, and also attenuated PKCβ2 and p38 MAPK signaling 28 days after STZ injection. All the effects of AT1 receptor blocker and tempol were mediated through the attenuation of both myocardial AT-II and oxidative stress, as evidenced from the reduction in the number of AT-II-positive cardiomyocytes and the reduced expression of p22 phox [130]. Collectively, these results suggest that AT-II *via* AT1 receptor and oxidative stress play a major role in diabetes-induced myocardial apoptosis, hypertrophy and fibrosis, and treatment with either an AT1 receptor blocker or an antioxidant will be beneficial for diabetic cardiomyopathy.

## SUMMARY

Diabetic cardiomyopathy is a clinical problem that develops in diabetes, and potentially involves oxidative stress, myocyte death, cardiac hypertrophy and fibrosis. These pathogenic changes may contribute to compromised ventricular dysfunction in diabetes, which is one of the leading causes of death in the world today. It is critical to investigate the underlying causes of diabetic cardiomyopathy and the synergetic effects of oxidative stress in combination with antioxidant therapy on the development of heart dysfunction-associated diseases. We have shown the signaling pathways of diabetes-induced myocardial apoptosis, hypertrophy, and fibrosis, and the multiple signaling pathways activated in diabetic cardiomyopathy are shown in Figs. (**1** and **2**). Studies are presently underway to identify the signaling pathways and oxidative stress in the development of diabetic cardiomyopathy.

## Figures and Tables

**Fig. (1):**
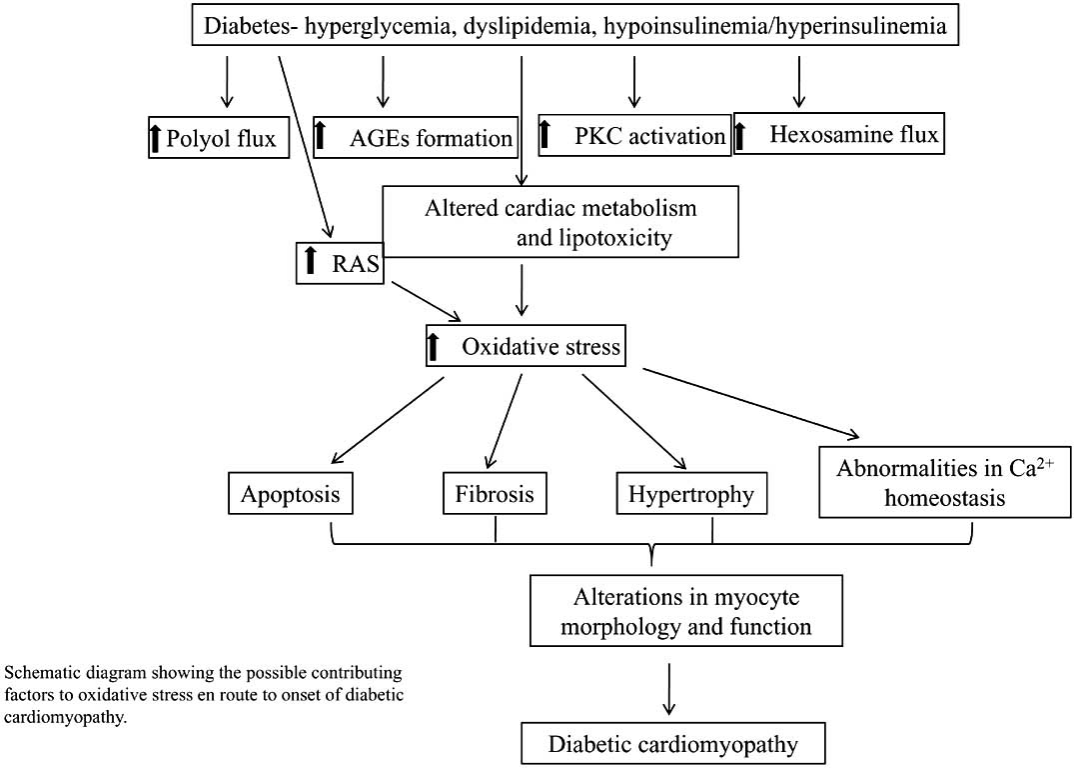
Schematic diagram showing the possible contributing factors to oxidative stress en route to onset of diabetic cardiomyopathy.

**Fig. (2):**
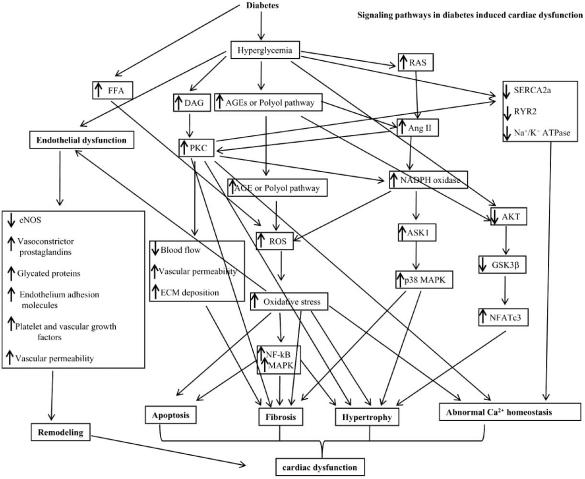
Signaling pathways in diabetes induced cardiac dysfunction.
